# A Second Chance: Managing Late Implant Failure from Peri-Implantitis with Computer-Guided Bone Regeneration—A Clinical Case Report

**DOI:** 10.3390/reports8030118

**Published:** 2025-07-22

**Authors:** Marco Tallarico, Silvio Mario Meloni, Carlotta Cacciò, Francesco Mattia Ceruso, Aurea Immacolata Lumbau

**Affiliations:** 1 Department of Medicine, Surgery and Pharmacy, University of Sassari, 07100 Sassari, Italy; smeloni@uniss.it (S.M.M.); alumbau@uniss.it (A.I.L.); 2 Department of Dentistry “Fra G.B. Orsenigo—Ospedale San Pietro F.B.F.”, 00189 Rome, Italy; f.m.ceruso@gmail.com

**Keywords:** computer-guided bone regeneration, titanium mesh, dental implants, bone regeneration, mandibular atrophy

## Abstract

**Background and Clinical Significance**: The retreatment of failed dental implants remains a challenging clinical scenario, particularly when complicated by peri-implantitis and as sociated bone loss. Successful management requires a comprehensive and predictable approach that addresses both hard and soft tissue deficiencies. **Case Presentation:** This case report illustrates a fully digital, prosthetically driven workflow for the rehabilitation of a posterior mandibular site following implant failure. A 44-year-old female patient underwent removal of a failing implant and adjacent tooth due to advanced peri-implantitis and periodontitis. After healing, a digital workflow—including intraoral scanning, cone-beam computed tomography (CBCT), and virtual planning—was employed to design and fabricate a customized CAD/CAM titanium mesh for vertical guided bone regeneration. The grafting procedure utilized a composite mixture of autogenous bone and anorganic bovine bone (A-Oss). After nine months of healing, two implants with a hydrophilic surface (SOI) were placed using a fully guided surgical protocol (OneGuide system). Subsequent soft tissue grafting and final prosthetic rehabilitation with monolithic zirconia restorations resulted in stable functional and aesthetic outcomes. **Conclusions:** This case highlights how the integration of modern digital technologies with advanced regenerative procedures and innovative implant surfaces can enhance the predictability and long-term success of implant retreatment in compromised posterior sites.

## 1. Introduction and Clinical Significance

Dental implants have become the gold standard for replacing missing or failing teeth since their introduction by Brånemark in the 1970s [1]. While implant-supported restorations offer predictable and effective outcomes, they are not without biological and mechanical complications. Reported failure rates range from 1% to 19% [2,3], and every implant treatment plan must consider these potential risks. Importantly, the decision to replace a compromised tooth with a dental implant requires careful evaluation. Pjetursson and Heimisdottir [4] proposed a classification system for natural teeth as secure, doubtful, or irrational to treat. Secure teeth are expected to function without complex intervention, while doubtful teeth may require advanced therapies and close maintenance. Teeth deemed irrational to treat are beyond salvage and are best managed through extraction. However, this classification can be subjective, and no universally accepted, evidence-based criteria currently define the so-called “failing dentition”. Moreover, alternative treatments may offer comparable outcomes to implant therapy. For example, endodontic retreatment of teeth with persistent pathology and questionable prognosis has shown similar success rates to implant rehabilitation [5]. Likewise, regenerative periodontal therapy can alter the prognosis of hopeless teeth, often providing a more cost-effective solution than extraction and implant placement [6]. Ultimately, no implant can exceed the longevity of a healthy, well-maintained natural tooth. Implant failures are generally categorized as early or late, depending on whether they occur before or after functional loading [7]. Early failures occurred before the application of functional loading, while, late failures occurred after applying occlusal loading or the first removal of the provisional restoration. Early failures, often related to the inability to achieve osseointegration, are primarily biological in origin [8,9]. In contrast, late failures can arise from either biological or mechanical complications. Peri-implantitis, a common biological issue, leads to the progressive loss of peri-implant hard and soft tissues [10,11]. Mechanical complications, such as overload or prosthetic misfit, may result in fractures of the implant body, abutment screw, or prosthetic components [12,13]. Managing late implant failure is especially challenging. These cases typically involve significant bone loss and occur after prosthetic treatment has been completed, often leading to patient dissatisfaction due to added costs and extended treatment time [14]. Among late failures, peri-implantitis is one of the most prevalent and difficult conditions to manage, often requiring complex retreatment protocols. Retreatment in such scenarios carries a higher risk of failure, partly because underlying risk factors may persist, and bone deficiencies often necessitate advanced regenerative procedures. The key point is to evaluate not only the staging of the pathology but particularly the grading or progression, which are both related to the age and medical condition of the patients.

One promising solution is computer-guided bone regeneration (CGBR), which combines computer-aided design (CAD) and manufacturing (CAM) of customized titanium mesh, together with a prosthetically driven workflow, to preoperatively plan and execute precise bone augmentation based on the intended prosthetic outcome [15,16].

This case report presents a comprehensive, fully digital approach to the aesthetic and functional rehabilitation of a failed dental implant due to peri-implantitis. Vertical GBR was needed due to the amount of bone loss. It highlights the complexity of managing late implant failures and demonstrates how modern digital tools, particularly within the framework of CGBR, can improve treatment planning and outcomes. Furthermore, it underscores the importance of a prosthetically driven approach in reducing complications and enhancing the success of retreatment in previously failed implant cases.

## 2. Case Summary

**Figure 1:**
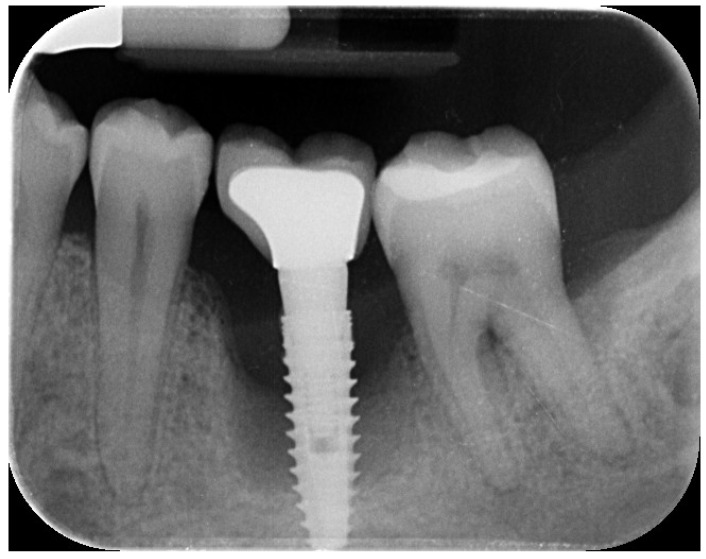
Initial radiographic assessment showing peri-implantitis on the implant and periodontal involvement on the second left mandibular molar.

A 44-year-old female patient presented with pain and swelling in the left mandibular region, primarily motivated by functional concerns and a desire to improve periodontal health. The patient reported the placement of a dental implant six years earlier in the region of the left mandibular first molar, which had replaced a hopeless tooth (Figure 1).

The patient was systemically healthy, a non-smoker, and diagnosed with localized advanced chronic periodontitis (AAP, 1999), including grade 2 mobility and grade III furcation involvement of the adjacent second left mandibular molar [17]. The probing depth around the implant and adjacent second left mandibular molar was ≥6 mm, with spontaneous bleeding and suppuration. The overall bleeding on probing and the plaque index were respectively 7% and 6%.

**Figure 2:**
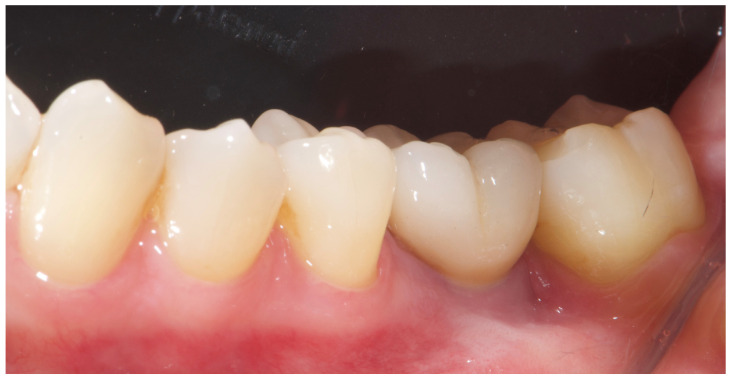
Initial clinical scenario showing gum inflammation and suppuration on the left mandibular first and second molars.

Clinical and radiographic evaluations revealed a cement-retained, implant-supported prosthesis at the site (Figure 2).

The implant site exhibited bleeding and suppuration upon gentle probing, probing depths ≥ 6 mm, and bone loss ≥ 3 mm apical to the coronal portion of the implant’s intraosseous segment. A diagnosis of peri-implantitis was made, which was consistent with the 2017 World Workshop criteria [18].

Treatment options—including retention or removal of the failing implant and molar—were discussed. Due to the patient’s age and extent of disease progression, the extraction of both the natural tooth and the implant was planned [19,20]. Written informed consent was obtained for all surgical and prosthetic procedures and the use of clinical and radiographic data for publication. The study adhered to the ethical principles of the 2013 Declaration of Helsinki.

On the day of surgery, the patient received antibiotic prophylaxis (2 g amoxicillin 1 h preoperatively, followed by 1 g twice daily for 6 days). The implant was atraumatically unscrewed and the tooth was extracted. Granulation tissue was thoroughly debrided to induce spontaneous bleeding, and a 4-0 resorbable suture was applied.

Eight weeks post-extraction, a CBCT scan and an intraoral scan (Medit i700, Medit Corp., Seoul, Republic of Korea) were obtained. A diagnostic wax-up was produced, followed by prosthetically driven virtual implant planning. Two implants were planned for the first and second molar sites, suitable for a screw-retained prosthesis, regardless of the existing bone volume.

**Figure 3:**
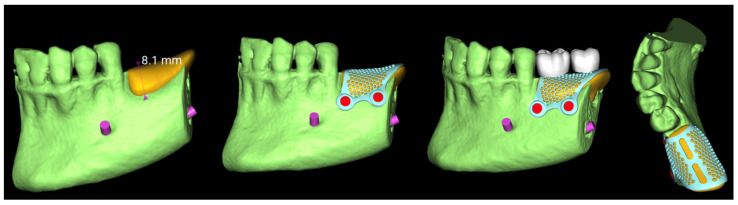
Vertical guided bone regeneration (vGBR) procedure. The amount of bone graft (in yellow) was evaluated and considered according to the prosthetic design (in white). The titanium mesh was designed accordingly.

The virtual implant planning was exported and a customized CAD/CAM titanium mesh was designed based on a commercially available template (OssBuilder, Osstem Implant Co. Ltd., Seoul, Republic of Korea) (Figure 3).

The mesh was designed by an expert using exocad (exocad’s PartialCAD 3.2 Elefsina, exocad GmbH, Darmstadt, Germany). and fabricated via laser melting (New Ancorvis Srl, Bargellino, Calderara di Reno, Italy). The maximum three-dimensional measures of the preplanned bone to be regenerated were 8.1 mm in height, 9.8 mm in width, and 19.6 mm in length, resulting in an estimated total volume of approximately 1197.8 mm^3^ (equivalent to 1.2 cm^3^). This volumetric estimation was derived from digital planning and served as a key parameter in guiding the grafting procedure and material selection.

A vertical guided bone regeneration (vGBR) procedure was performed by an experienced clinician (M.T.). Antibiotic prophylaxis (2 g amoxicillin 1 h preoperatively; 1 g twice daily for 8 days) was repeated. Prior to surgery, the patient rinsed with 0.2% chlorhexidine for 1 min, and the surgical site was isolated with a sterile drape. Local anesthesia was administered using 4% articaine with 1:100,000 epinephrine (Ubistein, 3M ESPE, Milan, Italy).

**Figure 4:**
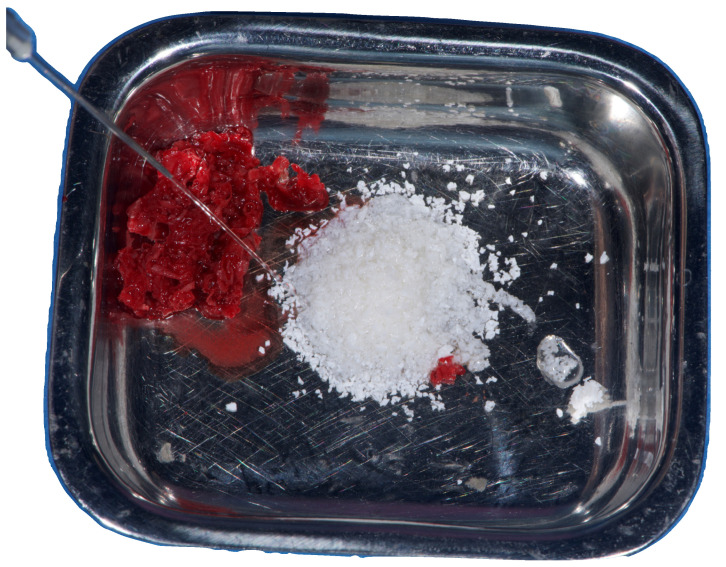
Bone regeneration materials: 50% of xenograft and 50% of autogenous bone collected using a cortical bone scraper.

**Figure 5:**
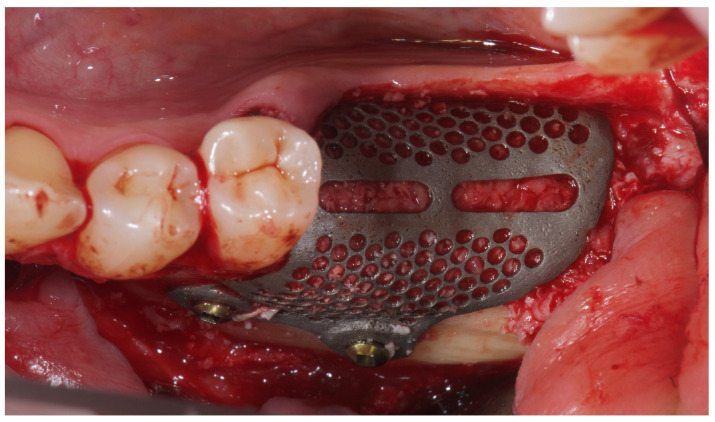
Titanium mesh placement using metal screws on the buccal cortical bone.

A buccally advanced crestal incision was made through the keratinized tissue (blade No. 15c), and a full-thickness flap was elevated. Vertical releasing incisions were placed mesially (two teeth away) and distally into the retromolar region. The site was debrided and autogenous bone was harvested using a cortical bone scraper (Safe Scraper, Micross, Meta, Reggio Emilia, Italy). After that, low-temperature plasma sterilization of the mesh was carried out. Subsequently, a 1:1 mixture (Figure 4) of autogenous bone and anorganic bovine bone, with a particle size ranging from 0.25 to 1 mm (1 g [2 boxes of 0.5 g [2 CC]]; A-Oss, Osstem Implant) was placed inside the titanium mesh and it was secured on the defect. The titanium mesh was fixed with two screws (Figure 5). Plasma treatment not only allows for high-level sterilization but also allows for a high level of decontamination, increasing the surface energy and the hydrophilicity of the titanium mesh [21].

The flaps were carefully mobilized, both buccally and lingually for tension-free closure, and 5-0 resorbable sutures were applied. Particularly, the lingual flap was carefully elevated to preserve the mylohyoid muscle and minimize trauma to surrounding tissues, potentially improving flap extension and healing [22].

**Figure 6:**
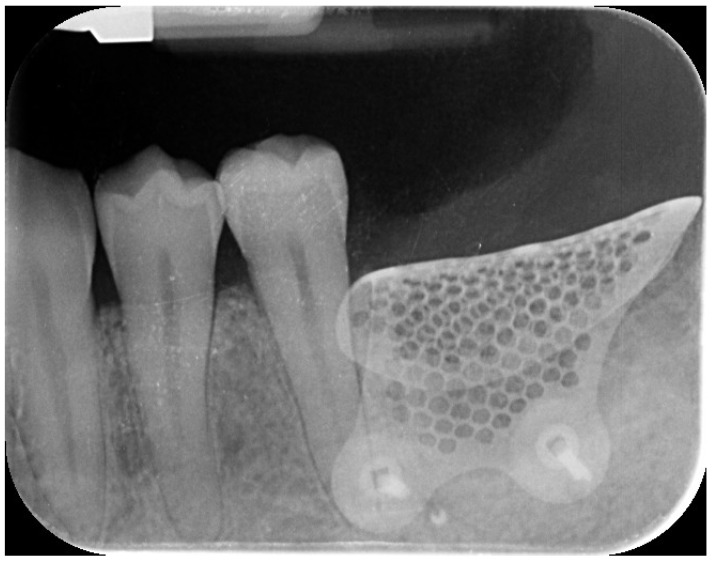
Postoperative intraoral radiograph.

**Figure 7:**
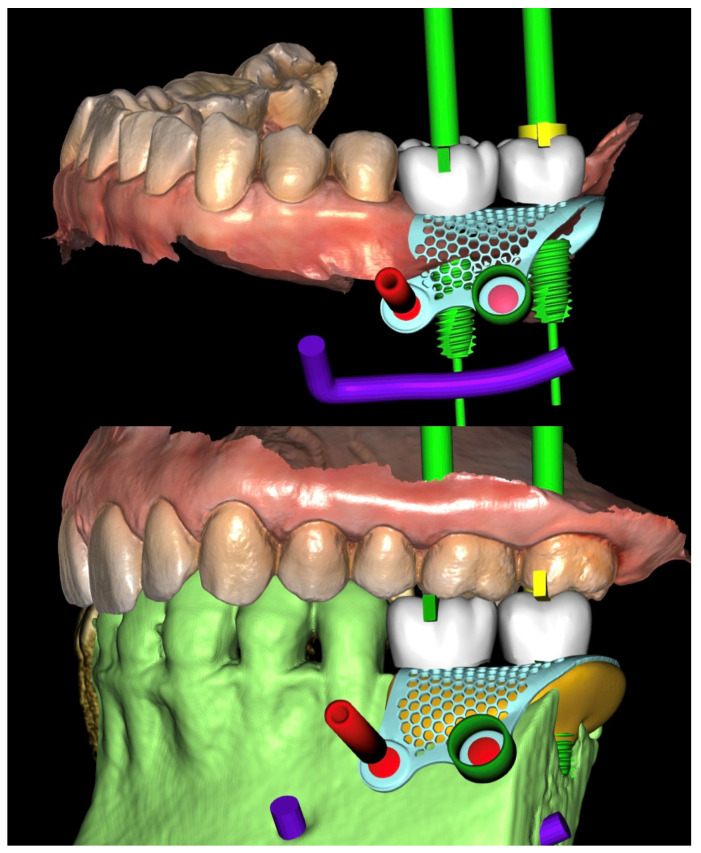
Virtual implant planning. The implant positions were planned according to the prosthetic wax-up.

Nine months later, a periodical radiograph (Figure 6) and clinical examination confirmed adequate bone regeneration. Two implants (Osstem TSIII SOI, 4.5 mm in diameter and 10 mm in length; Osstem Implant Co., Ltd., Seoul, Republic of Korea) with hydrophilic surfaces were placed under local anesthesia, using a fully guided (Figure 7), metal-sleeve-free surgical template (OneGuide, Osstem Implant Co., Ltd., Seoul, Republic of Korea).

**Figure 8:**
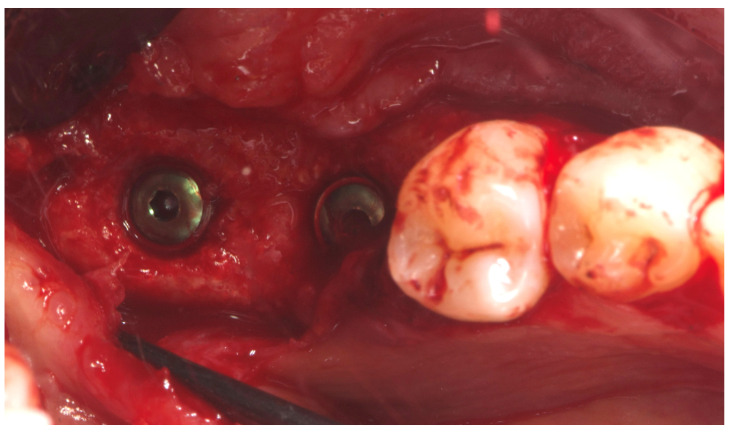
Second-stage surgery showing well-integrated implants and healthy regenerated bone.

Notably, the buccal portion of the titanium mesh was completely covered by newly formed cortical bone, allowing for only a partial removal of the mesh prior to implant placement (Figure 8).

**Figure 9:**
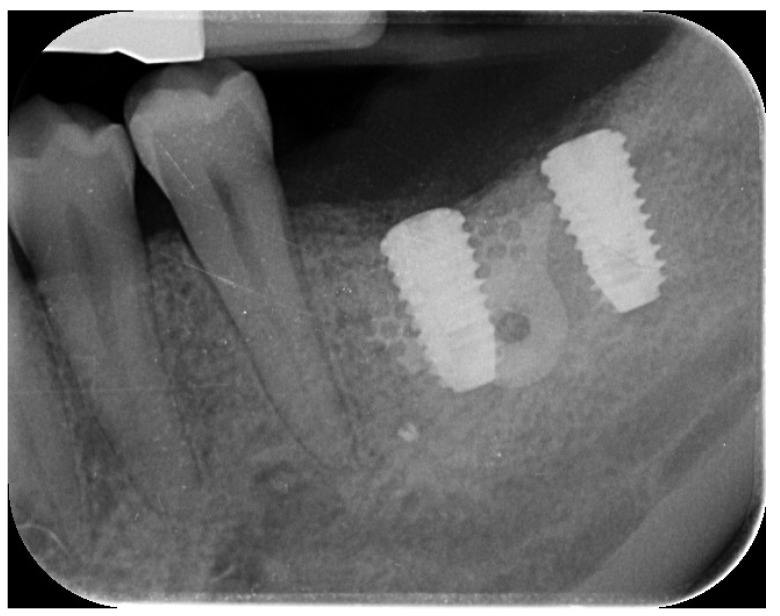
Radiographic assessment showing clear visibility of the buccal portion of the membrane left in situ.

Two months later, a second-stage surgery was performed. A horizontal incision was made between the keratinized tissue and mucosa, and a partial-thickness flap was apically repositioned (Figure 9).

**Figure 10:**
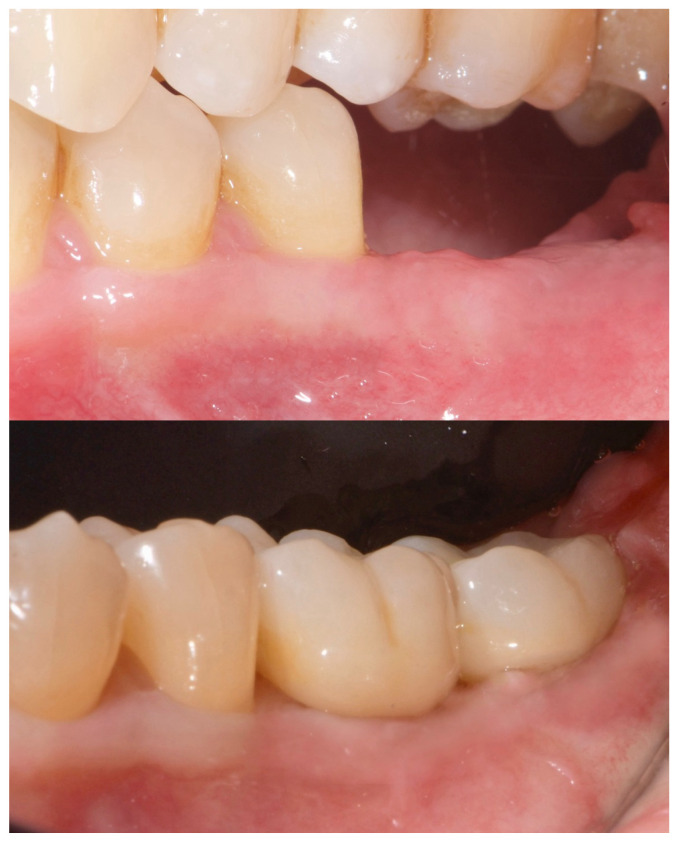
Before and after prosthesis delivery. Two single monolithic zirconia crowns were delivered and bonded to titanium link abutments, ensuring functional and aesthetic rehabilitation.

A free gingival graft (FGG), measuring 20 mm × 6 mm, was harvested from the palatal mucosa (first premolar to first molar). The epithelial layer of the FGG was partially removed to allow for partial subflap insertion, while a portion remained exposed. The graft was stabilized with resorbable single sutures. Two months post-grafting, a screw-retained temporary restoration was placed. Four months later, two single monolithic zirconia crowns, bonded to titanium link abutments, were delivered (Figure 10).

**Figure 11:**
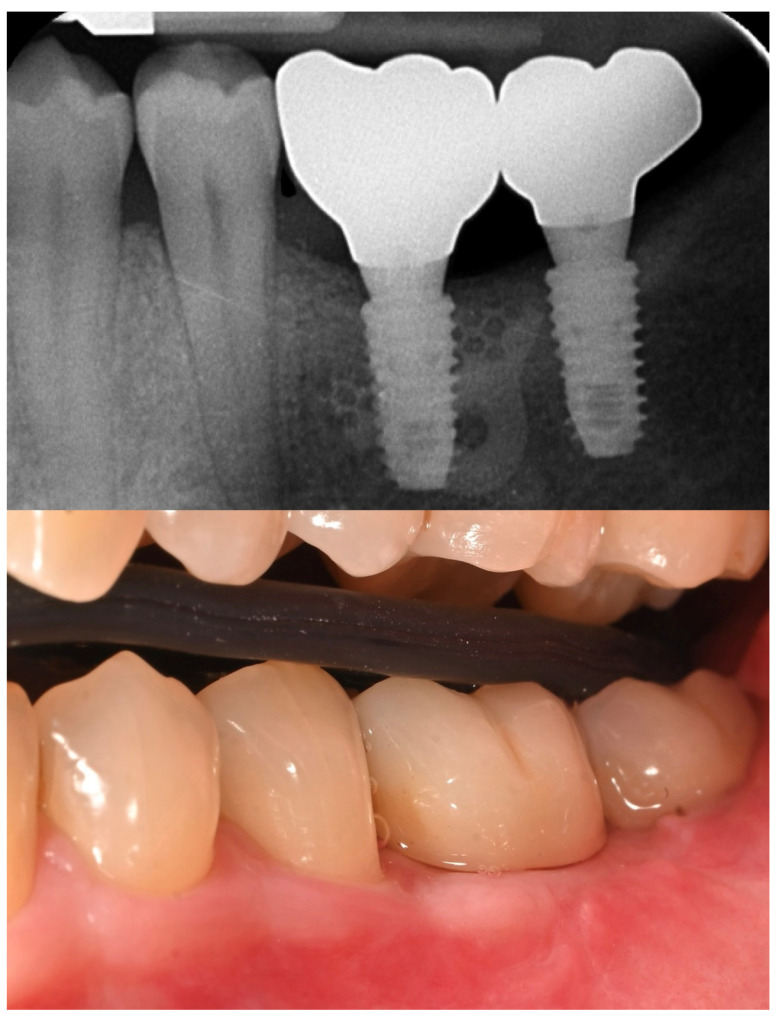
One-year radiographic and clinical follow-up.

The patient entered a 6-month hygiene maintenance protocol and continues to be monitored. One year after definitive prosthesis delivery, the implants were clinically stable, with no bleeding on probing. Radiographically, the bone remains stable, with no bone loss (Figure 11).

## 3. Discussion

The retreatment of failed dental implants presents a complex clinical scenario that demands careful diagnosis, patient-centered planning, and the use of advanced regenerative and prosthetic technologies. In this case, the decision to remove a compromised implant affected by peri-implantitis—along with the adjacent hopeless molar—was driven by progressive bone loss, high disease risk, and the patient’s functional and periodontal concerns. Peri-implantitis remains one of the primary causes of late implant failure, and its resolution often necessitates implant removal, especially when bone loss exceeds 50% of the implant length or regenerative predictability is low [10,11].

Rehabilitation in the posterior mandible after implant failure is further complicated by vertical and horizontal bone deficiencies that often follow explantation. Guided bone regeneration (GBR) remains the gold standard for such defects, but vertical augmentation remains technique-sensitive and unpredictable. In recent years, the emergence of computer-guided bone regeneration (CGBR) using CAD/CAM technologies has revolutionized this field, improving the precision and predictability of complex bone augmentation procedures [15,16]. The use of a customized titanium mesh, as in the present case, has shown promising outcomes in vertical ridge augmentation by stabilizing the graft and maintaining space for bone regeneration while conforming to the patient’s anatomy [22].

Specifically, the OssBuilder titanium mesh, designed and fabricated through a digital workflow, enabled ideal contouring of the graft volume while minimizing intraoperative adjustments. Tallarico et al. have reported favorable outcomes using customized titanium meshes in vertical GBR, emphasizing the benefits of digital planning, passive fit, and reduced surgical time [15]. In this case, digital planning was driven by prosthetic requirements and aided by intraoral scanning and CBCT merging. This approach aligns with the principles of prosthetically guided regeneration, ensuring that implant placement supports the intended restorative design from the outset.

The bone grafting protocol combined autogenous bone—harvested from the surgical site—with anorganic bovine bone (A-Oss) in a 1:1 ratio. A-Oss has been shown to promote osteoconduction and volume stability, particularly when used in combination with autogenous bone to enhance regenerative outcomes [23]. This biomaterial has also been validated in multiple GBR protocols in posterior mandibular defects, including those involving titanium mesh [15].

Following successful regeneration, implant placement was performed using a fully guided, metal-sleeve-free protocol (OneGuide, Osstem). This surgical system has been shown to improve accuracy in implant positioning and reduce intraoperative risk, particularly in anatomically challenging or regenerated sites. Tallarico et al. have documented the clinical reliability of guided surgery using OneGuide in combination with preoperative CBCT and digital wax-up protocols, confirming high levels of implant placement precision and restoration fit [24]. Nevertheless, clinicians should remain aware of potential complications associated with customized titanium meshes, particularly the risk of soft tissue dehiscence and early mesh exposure, which may compromise regenerative outcomes. While resorbable membranes offer a lower risk of exposure, they cannot be individually pre-shaped to the defect morphology and therefore lack the precision and volumetric stability provided by computer-guided bone regeneration. The ability to digitally design the mesh based on the patient’s anatomy remains a key advantage in achieving predictable, prosthetically driven outcomes in complex vertical defects.

The implants selected in this case featured the SOI (Surface Osstem Implant) hydrophilic surface, which is designed to enhance early osseointegration. Hydrophilic implant surfaces have demonstrated a superior biological response, particularly in compromised bone or grafted sites, by promoting rapid protein adsorption and early cellular adhesion [21]. Tallarico and colleagues have recently investigated this surface in challenging clinical cases, showing favorable short- and medium-term outcomes in both native and regenerated bone [15,21].

Overall, this case underscores the value of a fully digital, prosthetically driven workflow in managing late implant failure and supporting reimplantation in previously compromised sites. While retreatment inherently carries a higher risk of complications, the integration of CAD/CAM regenerative planning, modern implant surfaces, and guided surgical techniques can enhance both the efficiency and predictability of complex rehabilitation. Importantly, interdisciplinary collaboration and long-term maintenance protocols remain essential to ensuring sustained functional and aesthetic outcomes in retreatment scenarios. Concerning the surgical aspects of implant therapy, there are several key factors for the achievement of successful implants with good long-term stability. According to the recent literature, respect of the biological principles is essential to ensure bone and soft tissue stability [25,26,27].

The primary limitations of this single case report are its limited generalizability to the broader population and the absence of a control group, both of which are inherent to the case report study design. Further randomized controlled trials are necessary to obtain more definitive and generalizable outcomes.

Although the present case demonstrates successful outcomes at the one-year mark, long-term monitoring is essential to assess the stability of regenerated tissues, risk of peri-implant disease, and prosthetic integrity over time. Patient selection plays a critical role in the success of vertical augmentation procedures, with factors such as non-smoking status, good oral hygiene, thick gingival phenotype, and high compliance significantly influencing prognosis. Finally, while computer-guided bone regeneration involves higher upfront costs due to digital planning and customized components, its precision and reduced intraoperative time may justify the investment in complex or high-risk cases compared to conventional GBR techniques.

## Data Availability

The original data presented in the study are included in the article, further inquiries can be directed to the corresponding author.
